# Effect of Hop Varieties and Forms in the Hopping Process on Non-Alcoholic Beer Quality

**DOI:** 10.3390/molecules27227910

**Published:** 2022-11-16

**Authors:** Kinga Adamenko, Joanna Kawa-Rygielska

**Affiliations:** Department of Fermentation and Cereals Technology, Wrocław University of Environmental and Life Sciences, 51-630 Wrocław, Poland

**Keywords:** brewing, non-alcoholic beer, hop, fermentation technology, fermented beverages, beer chemistry

## Abstract

The aim of this study was to determine how the hopping technique affects the quality of non-alcoholic beer (NAB). A series of NABs were brewed and tested for basic physicochemical characteristics, profiles of selected volatile compounds, and microbial contamination. The brewing process yielded 13 experimental groups of beers, all of which had an ethanol content of <0.5%*v*/*v*. Among the batches brewed with ‘Marynka’ hops, the pellet form was found to provide the highest concentrations of hop-derived volatile compounds, whereas in the ‘Magnum’ groups, the extracts and whole hops proved superior. Humulene and caryophyllene were the primary volatiles in terms of quantity. All the brews were contamination-free—no microbes other than yeast cells were detected. Their microbiological purity was also supported by an assay of beer-defect indicators (volatile compounds), which only showed low levels of acetaldehyde, 1-propanol, 2-methylbutanol, and 3-methylbutanol. The hopping technique deployed was found not to affect the physicochemical parameters of NABs, but did have a significant impact on their volatile compound profile.

## 1. Introduction

The hop (*Humulus lupulus* L.) is a climbing plant belonging to the genus *Humulus* of the family Cannabaceae including *Cannabis sativa* L. and the species of the former Celtidaceae family [1]. This plant—more specifically, their female inflorescence—is one of the core and essential ingredients of beer (the others being water, malt, and yeast). It is mainly responsible for shaping the flavor and aroma of beer, giving it its distinct pleasant and long-lasting bitterness. In addition to shaping beer’s sensory characteristics, hops also act as a vital anti-bacterial and sporicidal agent that extends the shelf-life of beer—a quality mainly attributable to the humulones, lupulones, and polyphenol-type compounds they contain [2]. Beer gains most of its bitterness when the wort and hops are boiled together, triggering the isomerization of α-acids in the resin into iso-α-acids. Researchers have also identified other compounds in hops that are bitter in flavor, including tannins and polyphenols. However, unlike the measured, pleasant bitterness of the isomerized α-acids, theirs is a tart, astringent, tannin acridity. Hops owe their aroma predominantly to the oils in the lupulin sacs, particularly the myrcene, β-caryophyllene, β-farnesene and humulenes. However, the hop profile of beer has also been shown to be formed by the ethanol fermentation process mediated by yeast, as the hop-derived compounds are metabolized by the microbes. This is primarily attributable to the bioconversion of monoterpene alcohols and mono-/sesquiterpene hydrocarbons, as well as by aromatic volatile thiols and cell enzymes acting upon the aglucones in hops [3].

The quality and composition of hop constituents are a function of multiple factors: the hops’ variety, growing region, farming conditions, weather, and agro-technical processes [4,5,6]. However, the form of the hops also plays a role, and directly affects the quality of the final product. Brewery hops come in many different forms—not only whole cones, but pellets and extracts as well. Hop pellets are made by milling whole hops, then pressing the resultant powder, cooling it, and sealing it in air-tight packaging under an inert atmosphere to preserve quality. Hop extract is obtained by leaching specific ingredients with solvents such as ethanol or carbon dioxide, then concentrating the extract by evaporating the solvent [7]. 

The quality of fermented beverages is highly determined by the microbial strains used in the process, including the content of alcohol, extract, flavor compounds, color, and acidity [8]. Accordingly, using special yeast strains in the fermentative production of non-alcoholic beers can help preserve the metabolites responsible for the sensory profile. For example, *Lindnera jadinii* has been shown to be an interesting, unconventional yeast strain, which can produce pleasant banana aromas in non-alcoholic beers [9]. Yeast imparts beer its distinctive taste and aroma during fermentation. *Saccharomycodes ludwigii* is one of many specialty varieties of yeast that can be used to make non-alcoholic and low-alcohol beer (NABLAB). This species is unable to ferment maltose (the main brewing sugar generated during the mashing of wort), which means that ethanol production is almost completely halted if appropriate process parameters are maintained [10]. According to data from Research and Markets (2021) [11], the global non-alcoholic beer market continues to grow at a strong pace, and will serve consumers for at least the next 10 years. The global value of the market was 16.65 billion dollars in 2021, and is excepted to rise to 23.27 billion dollars by 2025, according to the most recent forecasts. Therefore, new research on brewing technology and beer quality would seem to be quite essential at this juncture. The most recent studies on non-alcoholic beer (NAB) tend to focus on consumer research, harnessing non-conventional microbes in NAB production, sensory analysis, health aspects, and various methods of brewing [12,13,14]. 

To the best of our knowledge, there are no papers in the literature that deal with the research problem of the present study—evaluating how the hopping technique affects NAB quality. For our experiment, we used two varieties of hops in three different commercially-sold forms: extract, pellet, and whole, each at two different bitterness levels: 40 and 60 IBU. This translates to a total of 13 non-alcoholic beers (including the no-hops control). The aim of the present study was to characterize the quality of the final products. Analyses encompassed basic physicochemical parameters (e.g. alcoholic strength, extract content, color), carbohydrate profiles, fermentation by-products, volatile compounds responsible for hop aroma, selected indicators (volatile compounds) of beer faults, sensory evaluation of the final product, and microbial contamination testing. The variety, amount, and form of the hops are all directly associated with NAB quality, including the ethanol fermentation process, its products, and the aroma profiles of the volatile compounds. 

It has been shown that the basic attributes that differentiate hop varieties are α-acids, β-acids, and essential oils. The unique combination of these features allows for the categorization of varieties. Typically, hops are classified as bitter or aromatic on the basis of the α-acids they contain. However, at present, the high content of not only α-acids, but also essential oils is also important, which is why there are varieties with double action [15,16]. In the experiments for the hopping technique, two varieties of hop were used: Marynka and Magnum. Earlier studies by other authors proved that these varieties are characterized by a high content of volatile compounds with an interesting aroma profile, at the same time providing the potential for the production of highly aromatic non-alcoholic beers. Both varieties have a high content of α-acids, making them a good material for the production of beers with a high degree of bitterness [17,18]. Obtained non-alcoholic beer had a high bitterness level (40 and 60 IBU). Recent studies show that producers of non-alcoholic beers are forced to keep up with the needs and interests of consumers. Adapting to such requirements is possible through continuous improvements and attempts to maintain the typical features of conventional beer [19]. Beers obtained as part of the experiment were produced in accordance with the assumptions for the classic English India Pale Ale style, for which The Beer Judge Certification Program (BJCP) determines the level of bitterness in IBU levels at 40–60 [20]. Unlike other bitter foods, high bitterness in beer may be desired by some consumers. It has been shown that an increased perception of bitterness does not always lead to a reduction in liking and consumption, and may even be a positive trait for some. Liking and consuming Pale Ale style beers is related to the search for a bitter taste experience, which in this case is not an aversive feeling at all [21]. The degree of bitterness is therefore an important factor influencing the consumption habits of consumers who are increasingly curious to try different tastes and styles of beer because they want to have a varied experience [22,23].

## 2. Results and Discussion 

### 2.1. Analysis of Basic Physicochemical Parameters

Table 1 shows basic parameters for the NABs: alcoholic strength, original wort extract (Ew), true extract (Er), real degree of fermentation (RDF), energy value, color, and pH. 

The results indicate that our process can be used to produce beer with alcoholic strength below 0.5%*v*/*v*. The actual RDF value correlates directly with the alcoholic strength—the higher the RDF, the higher the alcohol content of the final product. The RDF was found to range from 5 to 8%, and the true extract content in the final product decreased by an average 0.5%*w*/*w* against the wort (pre-fermentation). Beers produced fermentatively with a specialty strain of *Saccharomycodes ludwigii* can be made with low RDFs if several key conditions are met. In the first phase of the production, the malt-mashing process must be modified to minimize glucose production, prioritizing maltose and dextrins instead. Dextrin-rich beers are full-bodied, which is particularly desirable in NABs. The enzymatic apparatus of *Saccharomycodes ludwigii* has the ability to ferment glucose, but not maltose—the primary brewing sugar. In this, it differs from the traditional top and bottom fermentation brewing yeasts—*Saccharomyces cerevisiae* and *Saccharomyces pastorianus* [10]. Other factors that need to be controlled are: the original wort levels (which should be lower than in traditional beer production), the temperature, time of fermentation, and the number of yeast cells pitched into the wort prior to fermentation [24]. It has also been shown that apart from *Saccharomycodes ludwigii*, there are also other strains that do not have the ability to ferment maltose and can be successfully used in the production of non-alcoholic beers. The authors examined and confirmed the usefulness of such strains as *Cyberlindnera Saternus, Torulaspora delbrueckii* or, for example, *Mrakia gelida* [25,26,27]. In addition, the biological method of producing non-alcoholic beers, which uses special strains of yeast, can have a number of benefits in beer production. Compared to the traditional dealcoholization process, the obtained beers are characterized by a richer aromatic profile and are devoid of sensory defects. Moreover, this technology contributes to the improvement of the ecology in breweries, thanks to the significant reduction of the energy used and the reduction of the carbon footprint of this branch of the fermentation industry, contributing to the strategy of sustainable development [28].

We found that the color of non-alcoholic beers is determined not only by the variety of hops, but also their form and amount. For example, the addition of Magnum extract hops (ME40) significantly changed the color of the beer (by 6.5 EBC units or more). The most pronounced change in color—by 20 EBC units—was noted for Marynka whole hops (AC60). The higher levels of pellet and cones changed to color by 4 to 8 EBC units (the latter extreme was recorded for Magnum hop pellet). The color of beer is primarily shaped by the raw materials used during the brewing process, especially the malt. However, there is also a secondary determinant of beer darkness—the oxidation of polyphenols originating from malt and hops, most importantly proanthocyanidin oligomers and flavan-3-ol monomers [29]. Color is an extremely important feature of beer, directly related to its appearance, which determines the quality of this beverage. First of all, it depends on the amount of melanoidin compounds that are produced in the malting process, and the basic factor shaping the color of beer is the type of malt used. It has also been shown that the final color of the beer may also be influenced by other factors. It has been proven that the higher fermentation temperature conducted with *Saccharomyces cerevisiae* leads to higher absorbance beers as a result of melanoidin oxidation and browning. Other studies have shown that the color also depends on the yeast strain [30,31].

Also crucial for beer production is the pH level. The various metabolites produced by yeast during alcoholic fermentation—including low-pH organic acids—can decrease this quality control parameter [32]. The pH of NABs can also be significantly decreased by various additional ingredients, such as fruits or fruit juice [12]. Our study showed similar pH values across all of the brewed NABs. Likewise, no differences were noted between calorie content of the beers, which averaged 27 kcal/100 mL beer. The energy value of beer depends on the type of this beverage, and therefore on the brewing technology and raw materials used for the production. It has been shown that the energy content of beer can range from 160 kcal/500 mL in lagers to 420 kcal/500 mL in strong styles such as Barley Wine. Beers with low alcohol content and beers with a low extract content have been proven to have an energy value of approximately 140 kcal / 500 mL [33]. Moreover, it has been shown that consuming limited amounts of beer with low energy value and low alcohol content does not adversely affect human health and does not significantly increase daily energy consumption [34]. 

### 2.2. Assay of Hop-Derived Volatile Compounds

The finished NABs were tested for the content of 16 hop-derived volatile compounds, the results of which are presented in Table 2 and Table 3. 

The identified compounds included: one alcohol (1-hexanol), two monoterpene hydrocarbons (β-myrcene, trans-geranic acid methyl ester), one monoterpene alcohol (linalool), three sesquiterpens (trans-α-bergamotene, (E)-β-farnesene, cadina-1(10),4-diene), four sesquiterpenoids (β-selinene, α-selinene, nerolidol, humulene epoxide I), and five sesquiterpene hydrocarbons (copaene, caryophyllene, humulene, γ–muurolene, γ–cadinene). Out of all hop-derived volatile compounds assayed, the sesquiterpenoids: humulene and caryophyllene, were the most abundant, with mean levels in the beer at 91.5 μg/L and 25.0 μg/L, respectively. Literature studies have described the aroma of humulene as woody and musty, whereas caryophyllene has been characterized as oily, fruity, woody, and spicy [35]. Since these two compounds make up the bulk of the aromatic compounds, one might expect that they should also play the largest role in shaping the hop aroma profile of NABs. However, high levels of a given volatile component of hops do not have to necessarily translate to high odor intensity or vice versa—in fact, the odor detection threshold has been shown to play a part as well [36]. This has been demonstrated by Su et al. (2022) [35], who investigated Chinook and Cascade hops and found that the high levels of caryophyllene and humulene were offset by their low detection threshold, leading to reduced odor intensity of the two compounds. In contrast, trans-α-bergamotene contributed a strong flowery and sweet aroma, despite its much lower levels. A similar trend was observed for linalool, which was perceived as a citrus note. Despite the low concentrations of trans-α-bergamotene and linalool in our own experimental NABs, they could have been greater contributors to the hoppy aroma than caryophyllene and humulene. The literature offers examples of studies on volatiles contained in ‘Marynka’ and ‘Magnum’ hops and beers brewed with them [18,19,37]. However, none have investigated how hop-derived volatile compound profile of beer produced with these varieties changes in response to different forms of hops (extract, pellet, cones). 

The present study results show that the quality and quantity of hop-derived volatile compounds in NABs were determined not only by the variety and amount of hops, but by their form as well. The highest total quantity of these compounds (276.60 μg/L) was detected in the NAB brewed with ‘Marynka’ pellets (AP60). This group also had the highest levels of most individual compounds, with the exception of four: β-myrcene, copaene, humulene, and humulene exposide I. Similarly, ‘Magnum’ beer also had the highest levels of volatile compounds when brewed with pelleted hops. Interestingly, pelleted hops provided almost 13% more volatile compounds (total) in the ‘Marynka’ beers, whereas on the ‘Magnum’ side, it was the extracts and whole hops that performed better in this regard (57% and 64% more volatiles, respectively). 

In terms of individual compounds, all of the ‘Marynka’ groups (extract, pellet, cones) had higher levels of 1-hexanol and trans-geranic acid methyl ester compared with the ‘Magnum’. On the other hand, ‘Magnum’ hops provided higher concentrations of humulene and humulene epoxide I across all forms. Beers brewed with ‘Magnum’ and ‘Marynka’ pellets had the highest levels of: gamma-muurolene, naphthalene, γ-cadinene and cadina-1(10),4-diene). ‘Marynka’ pellets and ‘Magnum’ cones provided beer rich in β-myrcene and linalool, whereas peak coapene was found in beer brewed with ‘Magnum’ cones and pellets. High levels of other compounds (caryophyllene, trans-α-bergamotene, α-selinene, cadina-1(10),4-diene)) were found in the batches brewed with ‘Marynka’ pellets and cones. 

Several compounds were not detected at all in some of the beer variants. ‘Magnum’ cone beer (MC40 and MC60) was completely devoid of 1-hexanol. No trans-geranic acid methyl ester was detected in any of the pellet-hopped beers, including the ‘Magnum’ (MP40 and MP60). Copaene was absent from beer brewed with ‘Marynka’ cones (AC40 and AC60). As expected, more hoppy, highly bitter (60 IBU) beers were characterized by higher levels of the volatile compounds than beers with IBU = 40. The difference in total volatiles ranged from 13% (‘Magnum’ extract beer) to 245% in extreme cases (‘Marynka’ pellet beer). This correlation with bitterness held true for most of the individual compounds, with the sole exception of 1-hexanol in the ‘Marynka’ extract groups (AE40 and AE60) and the ‘Magnum’ pellet groups (MP40 and MP60). It has been shown that the content of hexanol in fermented beverages may be influenced by several complex factors, including lyase activity. Perhaps the higher content of α-acids in beer with a higher degree of bitterness, derived from the hop extract inhibited the alcohol dehydrogenases Adh6p and Adh7p, which are responsible for the reactions of hexanal reduction to hexanol [38,39,40]. However, the presented hypothesis would require confirmation in future, detailed research in the field of genetic engineering in order to investigate the influence of individual hop components on the activity of the above enzymes. Besides, the final content of hexanol in beer is influenced not only by the hopping process from which this compound is derived, but it can also be produced during the alcoholic fermentation process as a result of the metabolism of yeast cells. For example, the type and concentration of unsaturated fatty acids may also be responsible for the formation of hexanol in fermented beverages. Some of them may significantly contribute to the biosynthesis of this compound. It has been proven that the higher concentration of α-linoleic acid in the fermentation medium increases the amount of hexanol in the final product by up to 62% [41]. The evolution of the hop volatile compound profile of beer is a highly dynamic and complex process. The associated reactions are regulated in large part by the evaporation and chemical breakdown of heat-sensitive compounds as the wort is boiled with the hops. In addition, there are constant and major chemical changes occurring between the various volatile compounds in hops. For example, monoterpenes are susceptible to oxidative breakdown, forming various aromatic and oxidized terpenoids, including linalool [42]. Yeast also plays a significant role in the bioconversion of hop-derived volatile compounds via ethanol fermentation to generate new and different volatiles [43]. Furthermore, it has been shown that some chemicals in hops can be adsorbed to the hydrophobic yeast cells during fermentation, bound with yeast-cell-wall components, or can migrate to the foam layer. As such, the quantitative and qualitative composition of beer volatiles is constantly in flux, and is determined not only by the variety and amount of the hops, but also the various stages of the brewing process [44].

### 2.3. Microbial Contamination Testing 

**Figure 1:**
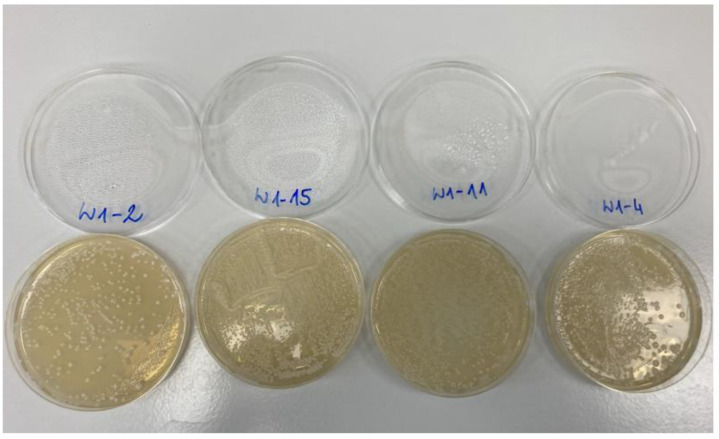
An example view of microbial cultivation in non-alcoholic beer. The labels correspond to the following batches: W1-2 is B0; W1-15 is ME40; W1-11 is AC40, W1-4 is AE60.

Our process for brewing non-alcoholic beer with *Saccharomycodes ludwigii* WSL17 produced microbiologically pure beverages. Apart from yeast cells, there were no bacteria or molds identified in the beers that would indicate contamination or spoilage. An indicative photograph of the Petri dishes with microbial cultures (grown in the experimental NABs) is provided in Figure 1. 

The fermentative production of NAB with *Saccharomycodes ludwigii* is complicated by the high risk of microbial contamination, due to the combination of low alcohol with high carbohydrates in the environment—prime conditions for growth of other, undesirable microorganisms. The beverage produced in our process was contamination-free, which was achieved by maintaining sterile conditions during yeast culturing, ensuring aseptic inoculation of the brewing wort, thoroughly washing and disinfecting all materials and equipment, and the proper hand-washing procedure on the part of the brewer. An interesting future challenge would be to find out how the antimicrobial properties of hops affect the quality of non-alcoholic beers. It has been proved that many hop-derived ingredients have been shown to exhibit antibacterial activity, which further protects the beer from infection. For example, a mixture of α-acids, β-acids, cohumulone, and/or columulone can inhibit most Gram-positive bacteria and some Gram-negative bacteria. Other bactericides in hops include: ferulic acid, humulinic acid, humulone, lupulone, and xanthohumol. In addition to their antibacterial properties, hops and their extracts can act as antifungal agents, inhibiting *Penicilium*, *Aspergillus*, and *Trichophyton* molds [45].

### 2.4. Quantitative, Qualitative, and Sensory Analysis of Volatile Compounds Indicative of Microbiological/Storage Defects 

The experimental batches were tested for volatiles that may, at higher levels, indicate microbial contamination and staling. Compounds were identified by way of GC-FID (methodology 3.6.) and sensory evaluation (methodology 3.7.). The beers were tested for eight key indicators (volatile compounds) of microbial contamination and three indicators of beer staling (Table 4). 

2,3-Butanedione (diacetyl) is a compound naturally formed during ethanol fermentation by yeast. It typically occurs in fresh beer, but can also indicate bacterial contamination at high concentrations. It is described as smelling of fresh butter, cream, or toffee. In the sensory tests, this compound was noted by only one panelist as occurring in the high-IBU Marynka-extract beer (AE60) (Table 5). 

GC-FID showed 2,3-butanedione to be present in all but the control group without hops (B0). Since the experimental beers were fresh (tested only a few days after brewing), the content of 2,3-butanedione was not attributed to microbial contamination, which was further confirmed by the findings in Section 3.4. Human sensitivity to 2,3-butanedione is 0.1–0.4 ppm [46]. [Krogerus and Gibson, 2013]. Trans-2-nonenal forms during beer aging, especially in the final stage of oxidation. It mostly affects the taste of light and pale beers (which include non-alcoholic beers) and leaves an aftertaste of wet cardboard, paper, and pencil. It was detected in AE60 and in AC40 (the IBU = 60 Marynka-cone beer) by one panelist each. 4-vinylguaiacol is another compound that can be generated by specialty or wild yeast, and is particularly desirable in wheat beers. However, it is considered a defect in most beer styles, as its presence can be caused by bacterial contamination. The scent of 4-vinylguaiacol is clove-like, spicy, and herbaceous. One panelist identified this compound in the high-IBU Marynka-pellet beer (AP60), whereas two others detected it in the IBU = 40 Marynka-cone (AC40) and the Magnum-extract (ME40) beers. Trans-2-nonenal and 4-vinylguaiacol were noted by the panelists is several beers, despite none being present according to the GC-MS. Acetaldehyde is present in all beers, as it is produced by yeast during alcoholic fermentation. It can exert a positive effect at low concentrations (6–8 g/mL), imparting a fruity aroma to the beer. However, excessive levels can cause bacterial contamination, producing green-apple and solvent aromas (similar to the smell of emulsion paint). The assessors identified acetaldehyde in most of the experimental NAB batches, including the low-IBU beer brewed with whole ‘Marynka’ hops (AC40), in which it was detected by all panelists. These findings are supported by the GC-FID analysis. The sensory detection threshold of acetaldehyde usually ranges from 5 to 20 mg/L. Fresh beer usually contains around 20–40 mg/L, which drops to 8–10 mg/L after maturation. P-menthane-8-mercapto-3-one is viewed purely as a beer defect (with the exception of new-wave hops, in which it occurs naturally) and is formed in early stages of oxidation. Its aroma resembles blackcurrants, kitty litter, and gooseberries. One panelist detected it in the IBU = 40 Magnum-extract batch (ME40), despite the fact that GC-MS failed to find it in any of the beers. Octanoic acid is produced by yeast during beer storage and can be an indicator of yeast autolysis. It is considered a defect in most beer styles. It has a waxy, goaty, or crayon aroma. Isovaleric acid can form as a result of bacterial contamination. It smells of fava beans, sweaty socks, or cheese. 2,4,6-trichloroanisole is a product of hyphae-growing fungi that enter into beer through outside contamination (from the packaging or raw materials). It is metabolized from chloroanisole and has a musty, moldy off-odor. No octanoic acid, isovaleric acid, or 2,4,6-trichloroanisole were identified by the assessors or by the GC-FID. 1-propanol, 2-methylbutanol, and 3-methylbutanol are higher alcohols, generated in the course of amino acid metabolism. In small amounts, they can add a fruity aroma to the beer; however, higher concentrations give out intense spirit and solvent aromas, which are considered an off-odor, especially in LABs. They can form as a result of microbial contamination. As seen in the table, one panelist found all of these compounds in five of the beers. However, GC-FID analysis detected the three compounds in all of the experimental groups. Higher alcohol levels in beer vary, usually within the 5–100 mg/L range. Higher levels of ethanol translate to higher content of fusel alcohols. This is corroborated by our study, which shows that the produced NABs contained no more than 3–4 mg/L [47,48]. Sensory analysis relies on the detection skills of the panelists, which are susceptible to subjective factors (physiological and psychological determinants such as the health condition, time of day, mood, etc.) and objective factors (conditions of the evaluation). In addition, each panelist has different detection thresholds and differential thresholds for specific volatile compounds. The sensitivity threshold for a particular aromatic compound is therefore an individual characteristic, which can affect the overall results of an analysis performed by a given group of people [49]. 

## 3. Materials and Methods

### 3.1. Plant and Biological Material

The wort was made from milled Pale Ale malt produced by Castle Malting (Beloeil, Belgium), as well as hops of two varieties (‘Marynka’ and ‘Magnum’) bought as pellets and whole cones from Centrum Piwowarstwa (Opole, Poland). The hop extracts of the same varieties were purchased from the company Powiśle (Wilków, Poland). According to the product data sheets, the hop extracts were produced using supercritical CO_2_ extraction. The malt was stored in a dry location prior to the experiment. All types of hops were kept refrigerated. The wort was fermented with *Saccharomycodes ludwigii* WSL17 yeast purchased as agar slant cultures from Hefebank Weihenstephan GmbH (Au i. d. Hallertau, Germany), kept at 8 °C before the culture process. 

### 3.2. Wort Preparation (Pre-Fermentation)

The malt was first mashed in 13.3 kg water. All of the beer variants were made with a 1:1 mixture of tap and distilled water. Distillation was done in an HLP10sp distillation device manufactured by Hydrolab (Straszyn, Poland). Starting water was deionized and amended with CaCO_3_ (1%), CaCl_2_ (1%) and CaSO_4_ (1%) to achieve the optimal water profile for Pale Ale production. Water pH was measured at 5.3 prior to mashing and acidified with 80% lactic acid. The pH was measured with a SevenCompact S220 pH-meter manufactured by Mettler Toledo (Greifensee, Switzerland). 

The water was then heated to 74 °C, and 3.8 kg of grist was added. Once the temperature was set and maintained at 72 °C, the mashing process was initiated and the mashbill continuously stirred. The products of this process (starch hydrolysis) were iodine–starch tested. A small amount of mash was sampled with a Pasteur pipette and plated onto the well of a porcelain plate. Iodine solution (2–3 drops, 0.01 M) was applied to the sample to evaluate presence of starch, repeated at 10 min intervals. After 30 min from the start of mashing (72 °C), the test was negative (no blue-purple color was formed). This process was continued for 5 min after which the temperature of the mash was raised by 1 °C/min to 78 °C, then kept at this temperature for 5 min (mash out). The entire mashing process lasted for 46 min.

The mash was then lautered with 23 L of water heated to 78 °C with pH = 5.8 (adjusted with 80% lactic acid). This process was carried out entirely in laboratory conditions. The mash was lautered through a braided filtration hose in a fermentation tank. After the lautering, the resultant wort was analyzed and adjusted to 8°Bx. The extract was measured at 20 °C throughout the experiment using a PAL-1 digital refractometer manufactured by Atago (Tokyo, Japan). 

The filtrate was divided into 13 experimental groups of 1.5 L each in duplicate. The classical hopping technology in this study was used where hops were added immediately after heating the wort to 100 °C. The process of boiling the wort with hops was conducted for the 60 min. In the experiment, the obtained beers had a bitterness level of 40 and 60 IBU. The value of bitterness, and thus the amount of added hops, was calculated on the basis of the α-acid content, declared by the producer of extracts, pellets and hop cones. In the Marynka variety, the α-acid content was: 44.4% in the extract, 7.9% in the pellet and 7.8% in the cone. In turn, in the Magnum variety: 44.9% in extract, 10.6% in pellets and 11.7% in cones. In order to calculate the dose of hops (in grams) for the wort boiling process, the following formula was used:

$$X\left(g\right)=\frac{\mathrm{IBU}\times 10\times V}{\alpha -\mathrm{acids}\;\times U}$$

where:

V—volume of the wort (L)

U—hop utilization: 30% for extract, 25% for pellet, 20% for coin

The experiment encompassed a no-hops control (B0), 6 groups brewed with ‘Marynka’ hops in different forms—extract: IBU = 40 (AE40) and IBU = 60 (AE60); pellet: IBU = 40 (AP40) and IBU = 60 (AP60); whole cones: IBU = 40 (AC40) and IBU = 60 (AC60); and 6 corresponding groups for the ‘Magnum’ hops—extract: IBU = 40 (ME40) and IBU = 60 (ME60); pellet: IBU = 40 (MP40) and IBU = 60 (MP60); and whole cones: IBU = 40 (MC40) and IBU = 60 (MC60). All variants were boiled at 100 °C for 60 min. After brewing, the water losses due to evaporation were measured and replenished with boiling water to the original volume (1.5 L). 

Hot wort (1.5 L) from each variant was poured into washed and sterilized 2 L glass bottles retrofitted with a set of hoses to vent the CO_2_ produced during the fermentation process, as well as another set of hoses to enable sampling without opening the bottles. All experimental variants were left to cool at 17 °C before microbial inoculation. 

### 3.3. Pre-Fermentation Yeast Culturing

A culture was set up to obtain enough yeast for the fermentation of the wort (produced using the process described in 3.2.). A YM medium was prepared, consisting of: yeast extract (3 g/L), malt extract (3 g/L), peptone (5 g/L), and glucose (10 g/L). All ingredients were dissolved in a set amount of distilled water. The pH of the medium was adjusted to 5.4, after which the medium was poured into three 10 mL test tubes, three 100 mL Erlenmeyer flasks, and three 1 L Erlenmeyer flasks. All test tubes and flasks were sealed with lignin stoppers and placed in an ASL autoclave from SMS (Warsaw, Poland) for 20 min at 121 °C. Once the media cooled down, a culture was performed under sterile conditions in an inoculation chamber and in the presence of a burner flame. The biological material was collected with a microstreaker and suspended in the media contained in the tubes. Thus inoculated, the tubes were cultured in a laboratory incubator from Binder (Tuttlingen, Germany) at 29 °C for 24 h. The suspension from the tubes was then transferred to the flasks containing 100 mL of the medium. Here, the yeast was grown for 24 h at 29 °C in a 357 shaking water bath from Elpin+ (Minsk Mazowiecki, Poland). Finally, the cultures were expanded into flasks with 1 L of the medium and grown for further 24 h under the same conditions. 

### 3.4. Fermentation of Wort 

The cultured yeast cells were counted using a Thoma chamber. A suspension of yeast cells was sampled for counting, diluted 10-fold in sterile distilled water, and mounted under a coverslip. After 5 min, the yeast was counted against the smallest squares of the chamber through a Leica ICC50 HD microscope (Wetzlar, Germany). To ensure accurate quantification, the cells were counted in 3 separately prepared microscope slides, with yeast counts being no less than 700. Cell counts per 1 mL of the culture were calculated according to the formula:

X = a·N·4·10^6^

where:

a—dilution factor

N—average cell count in the smallest square of the Thoma chamber

X = 10·33·4·10^6^ = 8·10^8^

The volume of suspension needed to inoculate 1.5 L of 8°Bx wort was then calculated, assuming minimum cell counts of 1·10^6^/mL wort/1°Bx. Per the calculations, 7.7 mL of suspension was taken from the obtained yeast culture and used to inoculate the wort with a sufficient microbial load. 

The wort was fermented at 17 °C for 72 h, then at 8 °C for 24 h. The final beer batches were bottled in 100 mL bottles and subjected to further analysis as part of the experiment. 

### 3.5. Analysis of Basic Physicochemical Parameters

The wort extract and real extract contents, real degree of fermentation, concentration of ethyl alcohol, and energy value were measured using an Anton Paar DMA 4500 M oscillating densitometer (Graz, Austria). Alcohol content was measured via near infrared (NIR) spectroscopy. Other parameters were calculated from the density value, which was measured by an oscillating U-tube. The beers were first degassed on the laboratory shaker 358A from Elpin+ (Mińsk Mazowiecki, Poland), filtered on laboratory filter paper, and subjected to analyses. Their pH value was measured with the pH-meter described in the Section 3.2. Energy value was calculated on the basis of gravity (*ρ*), real extract (Er), and alcohol content (A), according to the equation below. Analyses were carried out in duplicate.

Cal [kcal/100 mL] = (7 × A_[%*w*/*w*]_ + 3.5 × Er_[%*w/w*]_ × *ρ*_sample_)

### 3.6. Assay of Hop-Derived Volatile Compounds

Volatile compounds were analyzed by gas chromatography combined with a flame ionizing detector (GC-FID). Standards purchased from the Sigma-Aldrich company (Saint Louis, MO, USA) were used in this study for hop volatile compounds analysis: 1-hexanol (>99.5%), β-myrcene (>90%), linalool (>97%), trans-geranic acid methyl ester (>99%), copaene (>90%), trans-α-bergamotene (>90%), (E)-β-farnesene (>90%), humulene (>90%), γ-muurolene (>90%), β-selinene (>90%), α-selinene (>90%), γ-cadinene (<97%), cadina-1(10),4-diene (>90%), nerolidol (>97%), and humulene epoxide I (>96%). A GC2010 Plus gas chromatograph with a FID-2010 detector and a headspace autosampler (HS-20) (Shimadzu Corporation, Kyoto, Japan) equipped with a CP-WAX 57 CB column (50 m × 0.32 mm ID × 0.2 µm) (Agilent Technologies, Santa Clara, CA, USA) were used for the analysis, following a modified procedure by Kłosowski & Mikulski (2010) [50]. Samples were degassed, mixed with diatomaceous earth (1 g/100 mL), and filtered through the paper filter. Afterwards, 10 mL of the filtered beer were transferred to a 20 mL headspace vial. Each vial was conditioned in a headspace autosampler oven set to 40 °C and equilibrated for 20 min at a shaking level 2 prior to the injection of the sample into the column. Injection parameters were as follows: volume of the volatiles in the column = 1 mL, pressurizing time = 0.5 min, pressurizing equilibration time = 0.1 min, load time = 0.5 min, load equilibrium time = 0.1 min, injection time = 0.5 min, needle flush time = 0 min, total GC cycle time = 60 min. Injection mode was set to split (split ratio 10). The GC temperature program was as follows: 40 °C, hold 3 min, increase to 80 °C at a rate of 5 °C per min, hold 3 min, increase to 140 °C at a rate of 10 °C per min, hold 9 min, increase to 160 °C at a rate of 20 °C, hold 4 min (total program time 34 min). Column settings were as follows: starting pressure = 100 kPa, starting flow = 6.6 mL/min, starting column flow = 0.33 mL/min, starting linear velocity = 11.8 cm/s, purge flow = 3 mL/min. The carrier gas was helium. The FID operated at 280 °C at a sampling rate of 40 ms with the stop time at 34 min. The H_2_ flow to the FID was 50 mL/min, air flow was 400 mL/min, and makeup gas (helium) flow was 30 mL/min. Data were integrated and quantitated in the LabSolutions software (Shimadzu Corporation, Kyoto, Japan). Automatic integration was performed with the following conditions: peak width = 3 s, slope = at least 1000 uV/min, min. area = 1000 counts. Identification of the compounds was performed using analytical standards, with the identification method based on absolute retention time. Quantitation was performed using external standards, with five calibration points (coefficient of determination R² = at least 0.999).

### 3.7. Sensory Evaluation

The test consisted of a sensory evaluation to detect any volatile compounds potentially indicative of beer faults (spoilage during production and/or storage). The test was geared towards compounds produced by undesirable microbes, as excessive levels usually indicate microbial contamination The beer was evaluated for the content of: 2,3-butanedione, trans-2-nonenal, 4-vinylguaiacol, acetaldehyde, p-menthane-8-mercapto-3-one, octanoic acid, 2,4,6-trichloroanisole, isovaleric acid, 1-propanol, 2-methylbutanol, and 3-methylbutanol. The evaluation was performed by three experts with regular training on sensory analysis of beer, specializing in detecting beer faults. The panelists were instructed to indicate whether a given compound was detectable in the beer. 

### 3.8. Microbial Contamination Testing

The beer was tested for microbial purity using the spread plate method on YM solid agar Petri dishes. The YM consisted of: yeast extract (3 g/L), malt extract (3 g/L), peptone (5 g/L), glucose (10 g/L), and agar (20 g/L). The ingredients were diluted in distilled water and heated to liquefy the agar. The medium was pH-adjusted to 6.9 and sterilized according to methodology 2.2.2. After autoclaving, 20–25 mL of the heated medium was poured onto Petri dishes. Once solidified, the medium was inoculated with 0.1 mL of beer onto the dish and spread with a sterile spatula on the agar. The plates were cultured in a laboratory incubator at 30 °C for 48 h. After incubation, the resultant microbial colonies were read macroscopically. 

### 3.9. Statistical Analysis 

Data were analyzed using Statistica 13.5 software (StatSoft, Tulsa, OK, USA) based on ANOVA (α = 0.05). Duncan’s test was used to analyze differences between mean values (*p* < 0.05). The tables show values of standard deviation.

## 4. Conclusions

The quality of the hop-derived volatile compounds is highly determined by the brewing process, including the variety, amount and—notably—the form of the hops. Beers with pelleted hops had a higher content of the analyzed compounds than the extract or whole-cone batches. Further research is needed to test this relationship in other selected hop varieties, with a view to fine-tune the hopping process and produce non-alcoholic beers (NABs) with a rich and interesting aroma profile. By maintaining a safe procedure during fermentative NAB production, we obtained beer that showed no signs of contamination, as indicated by microbiological analysis, gas chromatography, and sensory evaluation. Though the sensory evaluation was conducted by experts with regular training on sensory detection of defects, their assessment of the NABs differed from person to person and did not always correspond to the gas chromatography results. More in-depth studies are required that would combine sensory analysis with gas chromatography to devise a novel brewing process—one that would produce beers with low odor thresholds for the volatiles most attractive to consumers, as well as low levels of those indicative of brewing/microbiological/storage defects. Further experimental work is needed to analyze hops’ influence on the quality of non-alcoholic beer, which will concern the antimicrobial properties of this raw material in order to control shelf life and the colloidal or storage stability of this fermented beverage. 

## Figures and Tables

**Table 1:** Basic physicochemical parameters.

No.	Beer	Alcohol [%*v*/*v*]	Ew [%*w*/*w*]	Er [%*w*/*w*]	RDF [%]	Kcal/100 mL	Color [EBC]	pH
1	B0	0.24 ± 0.0 ^d1^	7.16 ± 0.13 ^efg^	6.78 ± 0.09 ^efg^	5.44 ± 0.48 ^c^	25.68 ± 0.49 ^def^	14.59 ± 0.88 ^f^	5.07 ± 0.01 ^a^
2	AE40	0.33 ± 0.04 ^bcd^	7.23 ± 0.07 ^efg^	6.73 ± 0.12 ^efg^	7.03 ± 0.94 ^abc^	25.93 ± 0.25 ^def^	22.25 ± 0.33 ^e^	4.84 ± 0.02 ^ef^
3	AE60	0.34 ± 0.06 ^bcd^	7.50 ± 0.32 ^cde^	6.99 ± 0.23 ^cde^	7.10 ± 1.00 ^abc^	26.94 ± 1.19 ^cde^	22.87 ± 1.18 ^de^	4.82 ± 0.00 ^f^
4	AP40	0.33 ± 0.03 ^bcd^	7.09 ± 0.34 ^efg^	6.59 ± 0.28 ^efg^	7.39 ± 0.43 ^a^	25.42 ± 1.24 ^def^	24.17 ± 1.35 ^cde^	4.89 ± 0.02 ^def^
5	AP60	0.33 ± 0.02 ^bcd^	6.76 ± 0.18 ^fg^	6.24 ± 0.18 ^fg^	7.74 ± 0.58 ^ab^	24.13 ± 0.66 ^ef^	28.21 ± 0.64 ^bc^	4.96 ± 0.03 ^bc^
6	AC40	0.46 ± 0.02 ^a^	8.40 ± 0.11 ^ab^	7.70 ± 0.14 ^abc^	8.67 ± 0.47 ^a^	30.25 ± 0.43 ^ab^	28.91 ± 0.62 ^ab^	5.02 ± 0.02 ^ab^
7	AC60	0.46 ± 0.05 ^a^	8.94 ± 0.43 ^a^	8.25 ± 0.36 ^a^	8.02 ± 0.41 ^a^	32.27 ± 1.60 ^a^	33.53 ± 2.29 ^a^	5.00 ± 0.06 ^b^
8	ME40	0.33 ± 0.05 ^bcd^	7.27 ± 0.06 ^egd^	6.77 ± 0.02 ^efg^	7.15 ± 1.03 ^abc^	26.07 ± 0.22 ^def^	21.11 ± 2.82 ^e^	4.90 ± 0.03 ^de^
9	ME60	0.28 ± 0.03 ^cd^	7.93 ± 0.37 ^bcd^	7.50 ± 0.33 ^bcd^	5.59 ± 0.27 ^bc^	28.52 ± 1.38 ^bcd^	22.57 ± 2.59 ^e^	4.84 ± 0.01 ^ef^
10	MP40	0.34 ± 0.03 ^bcd^	6.62 ± 0.24 ^g^	6.11 ± 0.21 ^g^	8.04 ± 0.57 ^a^	23.56 ± 0.72 ^f^	21.69 ± 0.85 ^e^	4.88 ± 0.03 ^def^
11	MP60	0.41 ± 0.02 ^ab^	8.18 ± 0.46 ^abc^	7.55 ± 0.43 ^abcd^	7.98 ± 0.19 ^a^	29.43 ± 1.70 ^abc^	29.45 ± 2.32 ^ab^	4.93 ± 0.02 ^cd^
12	MC40	0.42 ± 0.08 ^ab^	8.60 ± 0.21 ^ab^	7.97 ± 0.11 ^ab^	7.63 ± 1.19 ^a^	30.99 ± 0.80 ^ab^	23.65 ± 2.21 ^bcd^	5.00 ± 0.03 ^bc^
13	MC60	0.37 ± 0.07 ^abc^	7.48 ± 0.49 ^cde^	6.91 ± 0.38 ^def^	7.82 ± 1.08 ^a^	26.84 ± 1.82 ^cde^	27.55 ± 1.91 ^cde^	4.97 ± 0.01 ^bc^

^1^ Values are expressed as the mean (n = 2) ± standard deviation. Mean values with different letters (a, b, c, etc.) within the same column are statistically different (*p*-value < 0.05).

**Table 2:** Hop volatile compounds.

RT		4.524	6.791	8.933	12.968	13.961	14.7	14.877	15.162
Compound		1-Hexanol	β-Myrcene	Linalool	Trans-Geranic acid Methyl ester	Copaene	Caryophyllene	Trans-α-Bergamotene	(E)-β-Farnesene
No.	Beer	μg/L							
1	B0	nd ^1^	nd	nd	nd	nd	nd	nd	nd
2	AE40	1.78 ± 0.05 ^b2^	2.17 ± 0.06 ^h^	0.94 ± 0.03 ^f^	1.19 ± 0.03 ^e^	0.92 ± 0.02 ^e^	16.37 ± 0.44 ^cde^	1.19 ± 0.03 ^ef^	1.53 ± 0.04 ^cd^
3	AE60	0.93 ± 0.01 ^c^	6.01 ± 0.06 ^c^	1.46 ± 0.02 ^d^	2.42 ± 0.03 ^d^	1.24 ± 0.01 ^c^	25.55 ± 0.27 ^bc^	1.82 ± 0.02 ^cd^	2.51 ± 0.03 ^cd^
4	AP40	0.69 ± 0.02 ^a^	3.50 ± 0.09 ^g^	2.87 ± 0.03 ^b^	3.57 ± 0.09 ^c^	nd	13.35 ± 0.35 ^de^	1.21 ± 0.03 ^ef^	13.83 ± 0.37 ^b^
5	AP60	1.78 ± 0.11 ^b^	6.14 ± 0.02 ^c^	3.02 ± 0.04 ^a^	9.03 ± 0.44 ^a^	1.99 ± 0.02 ^b^	57.38 ± 1.66 ^a^	7.19 ± 0.51 ^a^	23.13 ± 0.06 ^a^
6	AC40	0.57 ± 0.02 ^d^	6.08 ± 0.17 ^c^	1.42 ± 0.05 ^d^	3.62 ± 0.10 ^c^	nd	11.17 ± 0.31 ^ef^	1.28 ± 0.04 ^ef^	0.24 ± 0.01 ^d^
7	AC60	1.22 ± 0.04 ^d^	9.12 ± 0.20 ^b^	1.73 ± 0.06 ^c^	4.05 ± 0.09 ^b^	nd	18.83 ± 0.23 ^cde^	3.35 ± 0.09 ^b^	6.71 ± 7.70 ^c^
8	ME40	1.76 ± 0.03 ^b^	3.86 ± 0.07 ^f^	0.87 ± 0.07 ^f^	0.53 ± 0.01 ^f^	1.02 ± 0.02 ^d^	28.00 ± 0.54 ^bc^	1.86 ± 0.04 ^c^	0.24 ± 0.00 ^d^
9	ME60	0.69 ± 0.01 ^b^	5.64 ± 0.07 ^d^	1.38 ± 0.08 ^d^	0.64 ± 0.01 ^f^	1.09 ± 0.01 ^d^	31.58 ± 0.40 ^b^	2.08 ± 0.03 ^c^	3.91 ± 0.05 ^cd^
10	MP40	0.91 ± 0.03 ^c^	0.84 ± 0.03 ^i^	0.20 ± 0.09 ^h^	nd	1.24 ± 0.04 ^c^	24.30 ± 0.84 ^bcd^	1.06 ± 0.04 ^ef^	1.43 ± 0.05 ^cd^
11	MP60	nd	4.65 ± 0.03 ^e^	1.07 ± 0.10 ^e^	nd	2.38 ± 0.13 ^a^	40.11 ± 1.39 ^bc^	1.51 ± 0.04 ^de^	1.96 ± 0.07 ^cd^
12	MC40	nd	8.94 ± 0.29 ^b^	0.78 ± 0.11 ^g^	0.38 ± 0.01 ^f^	nd	8.55 ± 0.28 ^ef^	0.41 ± 0.01 ^g^	0.40 ± 0.08 ^d^
13	MC60	nd	19.29 ± 0.34 ^a^	0.94 ± 0.12 ^f^	0.56 ± 0.01 ^f^	1.05 ± 0.02 ^d^	24.78 ± 0.44 ^bcd^	1.20 ± 0.02 ^ef^	0.94 ± 0.09 ^d^

^1^ non detected. ^2^ Values are expressed as the mean (n = 2) ± standard deviation. Mean values with different letters (a, b, c, etc.) within the same column are statistically different (*p*-value < 0.05).

**Table 3:** Hop volatile compounds cont.

RT		15.283	15.558	15.807	15.911	16.158	16.225	17.223	17.634
Compound		Humulene	γ-Muurolene	β-Selinene	α-Selinene	γ-Cadinene	Cadina-1(10),4-diene	Nerolidol	Humulene epoxide I
No.	Beer	μg/L							
1	B0	nd ^1^	nd	nd	nd	nd	nd	nd	nd
2	AE40	67.48 ± 1.81 ^e2^	2.21 ± 0.06 ^de^	2.74 ± 0.07 ^b^	2.43 ± 0.07 ^cd^	1.84 ± 0.05 ^d^	2.68 ± 0.07 ^d^	2.28 ± 0.06 ^e^	1.86 ± 0.05 ^d^
3	AE60	117.58 ± 1.23 ^c^	2.77 ± 0.03 ^c^	2.86 ± 0.03 ^b^	2.51 ± 0.03 ^bc^	2.80 ± 0.03 ^c^	3.75 ± 0.04 ^c^	2.31 ± 0.02 ^e^	1.84 ± 0.02 ^d^
4	AP40	30.64 ± 0.81 ^f^	1.53 ± 0.04 ^g^	1.59 ± 0.04 ^d^	1.26 ± 0.03 ^g^	0.86 ± 0.02 ^e^	1.25 ± 0.03 ^e^	3.61 ± 0.10 ^b^	nd
5	AP60	129.48 ± 2.86 ^b^	4.55 ± 0.23 ^a^	6.95 ± 0.27 ^a^	6.91 ± 0.38 ^a^	4.79 ± 0.32 ^a^	6.69 ± 0.43 ^a^	4.60 ± 0.10 ^a^	2.97 ± 0.26 ^c^
6	AC40	32.35 ± 0.89 ^f^	0.95 ± 0.03 ^h^	1.33 ± 0.04 ^e^	1.34 ± 0.04 f^g^	0.99 ± 0.03 ^e^	1.00 ± 0.03 ^e^	1.36 ± 0.04 ^h^	0.45 ± 0.01 ^h^
7	AC60	48.13 ± 1.05 ^f^	2.02 ± 0.01 ^ef^	2.84 ± 0.11 ^b^	2.20 ± 0.01 ^de^	1.89 ± 0.07 ^d^	2.52 ± 0.03 ^d^	2.44 ± 0.18 ^e^	0.89 ± 0.05 ^g^
8	ME40	120.80 ± 2.32 ^c^	2.39 ± 0.05 ^d^	2.36 ± 0.05 ^c^	2.15 ± 0.04 ^e^	2.76 ± 0.05 ^c^	3.70 ± 0.07 ^c^	2.44 ± 0.05 ^e^	2.97 ± 0.06 ^c^
9	ME60	132.28 ± 1.66 ^b^	2.77 ± 0.33 ^c^	2.71 ± 0.03 ^b^	2.29 ± 0.03 ^cde^	3.01 ± 0.04 ^c^	3.88 ± 0.05 ^c^	2.98 ± 0.04 ^d^	3.59 ± 0.05 ^b^
10	MP40	93.53 ± 3.24 ^d^	2.28 ± 0.08 ^d^	1.67 ± 0.06 ^d^	1.56 ± 0.05 ^f^	1.86 ± 0.06 ^d^	2.60 ± 0.09 ^d^	1.88 ± 0.07 ^f^	1.95 ± 0.07 ^d^
11	MP60	169.00 ± 5.40 ^a^	3.49 ± 0.22 ^b^	2.75 ± 0.08 ^b^	2.73 ± 0.11 ^b^	3.26 ± 0.17 ^b^	5.47 ± 0.16 ^b^	3.24 ± 0.14 ^c^	4.03 ± 0.15 ^a^
12	MC40	44.30 ± 1.45 ^f^	0.66 ± 0.02 ^i^	1.03 ± 0.03 ^f^	0.58 ± 0.02 ^h^	0.82 ± 0.03 ^e^	1.20 ± 0.04 ^e^	0.72 ± 0.02 ^i^	1.17 ± 0.04 ^f^
13	MC60	114.86 ± 2.03 ^c^	1.80 ± 0.03 ^f^	2.20 ± 0.04 ^c^	1.45 ± 0.03 ^fg^	1.92 ± 0.03 ^d^	2.56 ± 0.05 ^d^	1.59 ± 0.03 ^g^	1.52 ± 0.03 ^e^

^1^ non detected. ^2^ Values are expressed as the mean (n = 2) ± standard deviation. Mean values with different letters (a, b, c, etc.) within the same column are statistically different (*p*-value < 0.05).

**Table 4:** Volatile compounds indicative of beer faults.

Compound		2,3-Butanedione	Trans-2-nonenal	4-Vinyl guaiacol	Acetaldehyde	p-Menthano-8-mercapto-3-on	Octanoic acid	Isovaleric acid	2,4,6-Trichloroanizol	1-Propanol	2-Methylbutanol	3-Methylbutanol
No.	Beer	mg/L										
1	B0	nd ^1^	nd	nd	1.84 ± 0.04 ^bc^	nd	nd	nd	nd	0.85 ± 0.04 ^h^	0.68 ± 0.04 ^def^	1.68 ± 0.10 ^cde^
2	AE40	142.61 ± 2.60 ^ef2^	nd	nd	1.54 ± 0.03 ^d^	nd	nd	nd	nd	0.88 ± 0.02 ^h^	0.68 ± 0.01 ^def^	1.44 ± 0.00 ^g^
3	AE60	175.26 ± 11.45 ^c^	nd	nd	2.31 ± 0.13 ^a^	nd	nd	nd	nd	0.93 ± 0.01 ^fgh^	0.70 ± 0.01 ^de^	1.52 ± 0.06 ^fg^
4	AP40	135.64 ± 7.89 f^g^	nd	nd	1.74 ± 0.06 ^c^	nd	nd	nd	nd	0.91 ± 0.04 ^gh^	0.71 ± 0.01 ^d^	1.58 ± 0.04 ^efg^
5	AP60	436.85 ± 25.89 ^a^	nd	nd	1.50 ± 0.00 ^d^	nd	nd	nd	nd	1.04 ± 0.02 ^def^	0.69 ± 0.00 ^def^	1.46 ± 0.04 ^g^
6	AC40	116.52 ± 8.01 ^g^	nd	nd	1.58 ± 0.03 ^d^	nd	nd	nd	nd	1.18 ± 0.05 ^bc^	0.78 ± 0.01 ^c^	1.77 ± 0.02 ^bcd^
7	AC60	147.83 ± 10.22 ^def^	nd	nd	1.12 ± 0.03 ^f^	nd	nd	nd	nd	1.14 ± 0.06 ^bcde^	0.80 ± 0.02 ^bc^	1.80 ± 0.03 ^bc^
8	ME40	168.77 ± 9.38 ^cd^	nd	nd	1.54 ± 0.08 ^d^	nd	nd	nd	nd	0.92 ± 0.05 ^fgh^	0.65 ± 0.03 ^g^	1.55 ± 0.06 ^efg^
9	ME60	270.62 ± 8.72 ^b^	nd	nd	2.40 ± 0.07 ^a^	nd	nd	nd	nd	1.10 ± 0.09 ^cde^	0.66 ± 0.04 ^fg^	1.65 ± 0.09 ^def^
10	MP40	161.62 ± 5.72 ^cde^	nd	nd	1.25 ± 0.01 ^e^	nd	nd	nd	nd	1.01 ± 0.11 ^efg^	0.80 ± 0.04 ^bc^	1.68 ± 0.07 ^cde^
11	MP60	287.58 ± 5.66 ^b^	nd	nd	1.27 ± 0.04 ^e^	nd	nd	nd	nd	1.17 ± 0.00 ^bcd^	0.81 ± 0.01 ^bc^	1.83 ± 0.01 ^b^
12	MC40	74.59 ± 11.30 ^h^	nd	nd	1.90 ± 0.01 ^b^	nd	nd	nd	nd	1.35 ± 0.07 ^a^	0.92 ± 0.01 ^a^	2.11 ± 0.09 ^a^
13	MC60	71.00 ± 13.25 ^h^	nd	nd	1.89 ± 0.03 ^b^	nd	nd	nd	nd	1.27 ± 0.07 ^ab^	0.84 ± 0.01 ^b^	1.88 ± 0.01 ^b^

^1^ non detected. ^2^ Values are expressed as the mean (n = 2) ± standard deviation. Mean values with different letters (a, b, c, etc.) within the same column are statistically different (*p*-value < 0.05).

**Table 5:** Volatile compounds indicative of beer defects.

Compound		2,3-Butanedione	Trans-2-nonenal	4-Vinyl guaiacol	Acetaldehyde	p-Menthano-8-mercapto-3-on	Octanoic acid	Isovaleric acid	2,4,6-Trichloroanizol	1-Propanol	2-Methylbutanol	3-Methylbutanol
No.	Beer											
1	B0	nd ^1^	nd	nd	•	nd	nd	nd	nd	nd	nd	nd
2	AE40	nd	nd	nd	nd	nd	nd	nd	nd	nd	nd	nd
3	AE60	• ^2^	•	nd	•	nd	nd	nd	nd	•	•	•
4	AP40	nd	nd	nd	nd	nd	nd	nd	nd	nd	nd	nd
5	AP60	nd	nd	•	•	nd	nd	nd	nd	•	•	•
6	AC40	nd	•	••	•••	nd	nd	nd	nd	•	•	•
7	AC60	nd	nd	nd	••	nd	nd	nd	nd	nd	nd	nd
8	ME40	nd	nd	••	••	•	nd	nd	nd	nd	nd	nd
9	ME60	nd	nd	nd	nd	nd	nd	nd	nd	nd	nd	nd
10	MP40	nd	nd	nd	nd	nd	nd	nd	nd	nd	nd	nd
11	MP60	nd	nd	nd	nd	nd	nd	nd	nd	nd	nd	nd
12	MC40	nd	nd	nd	nd	nd	nd	nd	nd	•	•	•
13	MC60	nd	nd	••	•	nd	nd	nd	nd	•	•	•

^1^ non detected. ^2^ each dot denotes one person detecting the scent.

## Data Availability

Not applicable.

## Sample Availability

Samples of the compounds are available from the authors.
